# Complications After Ankle Fracture Surgery in Finland Between 1998 and 2020

**DOI:** 10.2106/JBJS.23.00745

**Published:** 2024-04-29

**Authors:** Ville Happonen, Heikki Kröger, Reijo Sund

**Affiliations:** 1 Department of Orthopedics, Traumatology and Hand Surgery, Kuopio University Hospital, Kuopio, Finland; 2 Kuopio Musculoskeletal Research Unit, University of Eastern Finland, Kuopio, Finland

## Abstract

**Background:**

Ankle fracture is a common injury and often requires operative treatment. This study investigated short-term (≤4 months) and long-term (>4 months) complications after ankle fracture surgery in a 23-year period with use of data from a comprehensive, nationwide, individual-level register.

**Methods:**

Data regarding patients who underwent operative treatment for ankle fracture were collected from the Finnish Care Register for Health Care and analyzed with use of logistic and Cox regression.

**Results:**

A total of 83,666 ankle fractures were operatively treated between 1998 and 2020. Of these, 36% were lateral malleolar fractures, 7% were medial malleolar fractures, 52% were bimalleolar or trimalleolar fractures, and 5% were other types of fractures. Fifty-one percent of the fractures were in female patients. The overall rate of short-term complications was 7.2%. Specifically, infection occurred in 4.4% of cases; thromboembolic complications,1.6% of cases; mechanical complications, 0.4% of cases; and other complications, 0.9% of cases. An age of >75 years was associated with a higher rate of short-term complications than an age of 51 to 75 years, with an odds ratio of 1.53 in the multivariable analysis (95% confidence interval, 1.39 to 1.67; p < 0.001). Short-term complications were also more prevalent in patients with diabetes (with or without associated complications); chronic pulmonary, kidney, or liver disease; or peripheral vascular disease. Mortality during the first 4 months after the ankle fracture operation was 0.6%. The most common reason for reoperation in the long term (>4 months after the index procedure) was fixation device removal, with a cumulative incidence of 17% within the first 3 years postoperatively. The risk of implant removal increased in younger patients and patients with bimalleolar or trimalleolar fractures. The cumulative incidence of ankle arthrodesis and arthroplasty was low.

**Conclusions:**

Although postoperative complications are relatively rare, their treatment can lead to considerable morbidity. The findings of this study allow us to identify patients who are prone to complications or reoperations after undergoing operative treatment for ankle fracture.

**Level of Evidence:**

Prognostic Level III. See Instructions for Authors for a complete description of levels of evidence.

Ankle fracture is one of the most common injuries requiring orthopaedic surgery^1^. The goal in the operative treatment of ankle fracture is an anatomical reduction and stable fixation that allows early mobilization and recovery of function. According to current recommendations, unstable ankle fractures should be treated with open or closed reduction and internal fixation. Research has shown that complications after ankle fracture surgery occur in up to 5% to 40% of cases^2^. Fracture type and patient-related risk factors (age, peripheral arterial disease, diabetes, obesity, smoking, alcohol use) can increase the risk of complications^3,4^. The most common early-term complications are wound complications, including infection; malreduction; loss of fixation; deep venous thrombosis; and pulmonary embolism. Long-term complications include delayed union or nonunion, pain related to the fixation device, and symptomatic ankle arthrosis^5-8^.

Our aim was to investigate mortality and early-term (≤120 days) and long-term (>120 days) complications following the operative treatment of ankle fracture.

## Materials and Methods

This comprehensive registry study encompassed the entire population of Finland, which comprised 5,533,793 residents as of December 31, 2020. We identified all patients who underwent inpatient or outpatient operative treatment for ankle fracture at Finnish hospitals between January 1, 1998, and December 31, 2020. Data were extracted from the Finnish Care Register for Health Care (CRHC, formerly known as the Finnish Hospital Discharge Register [FHDR]), a database maintained by the Finnish Institute for Health and Welfare. The dates and causes of deaths in this cohort were linked from the Causes of Death Register maintained by Statistics Finland. We gathered age- and sex-group-specific population sizes with use of StatFin, the online services of Statistics Finland.

The CRHC, which was established in 1967, is one of the oldest nationwide individual-level hospital discharge registers in the world. The CRHC offers information pertaining to the subject’s age, sex, place of residence, length of hospital stay, and primary and secondary diagnoses, as well as surgical procedures that were conducted during hospitalization. Data are extracted directly from the information systems of health-care providers and include all inpatient and outpatient admissions at public and private hospitals. The register consists of real-world data regarding all patients in the country. All admissions of a particular patient can be identified and linked across all health-care providers with use of the unique personal identity code. Diagnoses have been recorded with use of the International Classification of Diseases, Tenth Revision (ICD-10) since 1996. The Finnish version of the Nordic Medico-Statistical Committee (NOMESCO) procedure classification has also been utilized since 1996 to record surgical operations and procedures. The register has demonstrated good quality and validity in terms of its coverage and accuracy^9^.

In the present study, patients were identified from the register with use of diagnosis and procedure codes. There was no patient contact (e.g., through emails or telephone calls). Complications were identified from the CRHC with use of diagnosis and procedure codes that were consistent with hospital readmissions for the ankle fracture. We reviewed all S82.3 to S82.9 diagnoses and the associated surgical procedures and selected only the code combinations that referred to ankle fractures, as previously described^10,11^. Specifically, we employed ICD-10 codes S82.4 to S82.9 and NOMESCO procedure codes NHJ10 (internal fixation of ankle fracture), NHJ12 (internal fixation of ankle fracture using bioimplants), and NGJ70 (external fixation of lower-leg fracture). We categorized ankle fractures that were treated with use of specific procedures into 4 distinct classes: (1) medial malleolar fractures, identified by ICD-10 code S82.5; (2) lateral malleolar fractures, characterized by ICD-10 code S82.6; (3) bimalleolar and trimalleolar fractures, encompassing ICD-10 codes S82.7 to S82.8; and (4) other ankle fractures, encompassing ICD-10 codes S82.4 and S82.9.

Utilizing register-based data, we studied mortality and early and long-term complications. We reviewed all diagnoses that were recorded within 4 months (≤120 days) after the index ankle-fracture operation and selected the most commonly diagnosed complications, which we divided into 5 classes: infections, thromboembolic complications, mechanical complications (including nonunion), other complications, and mortality (Table I).

**TABLE I:** Classes and ICD-10 Diagnosis Codes of Short-Term (≤120 Days) Complications After Ankle Fracture Surgery

1. Infections
T81.4 postoperative infection not classified elsewhere
T84.68 infection and/or inflammatory reaction due to internal fixation
A41.0-A41.9 septicemia
M86.1 acute osteomyelitis
M86.9 unspecified osteomyelitis
T79.3 unclassified post-injury wound infection
2. Thromboembolic complications
I82.88 nonspecific embolism/thrombosis of vein
I82.9 unclassified embolism/thrombosis of vein
I26.0 pulmonary embolism and acute pulmonary heart disease
I26.9 pulmonary embolism without acute pulmonary heart disease
I80.29 phlebitis/thrombophlebitis of deep veins of the lower limbs
I80.3 unspecified lower-limb phlebitis or thrombophlebitis
3. Mechanical complications (including non-ossification)
T84.1 mechanical complication of internal fixation device of bones of limb
T84.4 mechanical complication of other internal prosthetic devices, orthopaedic implants, and grafts
M84.0 defective consolidation of fracture
M84.1 absent consolidation of fracture
M84.2 delayed ossification
4. Other complications
T93.2 late effects of other lower-limb fracture
T81.0 hemorrhage/hematoma after surgical or medical intervention
T81.3 wound rupture after the operation, not classified elsewhere
G57.4 disorder of the tibial nerve
G57.5 tarsal canal syndrome
M89.0 algoneurodystrophy, i.e., complex regional pain syndrome
5. Mortality

We also investigated NOMESCO procedure codes that referred to prolonged problems or complications in the long term (i.e., >120 days after the primary surgery). These codes consisted of NHG20 (arthrodesis of the ankle joint), NHB10 (prosthetic replacement of the ankle joint), NHA30 (endoscopic examination of the ankle joint or a foot joint), NHU20 (removal of the internal fixation device from the ankle or foot), NHJ10 (internal fixation of ankle fracture, either as a reoperation due to delayed union or nonunion or as primary operation for a new ankle fracture on the same side or the contralateral side), and NGQ20 (amputation of the lower leg or an amputation below the knee).

We stratified fractures by fracture type (medial malleolar, lateral malleolar, bimalleolar or trimalleolar, and other), age group (≤50, 51 to 75, and >75 years old), and sex. We examined patient comorbidities with use of the Charlson Comorbidity Index (CCI), which is a method of categorizing comorbidities on the basis of ICD diagnosis codes and was designed to predict 1-year mortality on the basis of a weighted composite score^12^. We included the CCI categories of diseases that were treated at hospitals in inpatient or outpatient settings within 2 years before the primary ankle fracture operation. The CCI has been adapted and validated to work with Finnish register data^13^.

### Statistical Analysis

The patient sample is described with use of descriptive statistics (Table II). We examined the cumulative incidence of short-term complications (mortality, infection, thromboembolic complications, mechanical complications, and other complications) within the 120-day follow-up period. We utilized univariate and multivariable logistic regression to analyze the risk of complications. Risks associated with patient-related covariates are expressed as odds ratios (ORs) relative to the reference group. P values and 95% confidence intervals (CIs) are reported as well. The level of significance was set at p < 0.05. We examined the cumulative incidence of long-term complications and utilized a time-to-event analysis with Cox proportional hazards regression models. The follow-up period for long-term complications began 120 days after the index operation and ended at the reoperation event, death, or the last day of the study period, whichever occurred first. In the model for late-complication reoperations that were performed without removal of the fixation device, the possibility of an earlier removal of the fixation device was handled by utilizing a binary time-dependent covariate. The strength of the association between the risk of reoperation in the long term and each variable is expressed as an adjusted hazard ratio relative to the reference group. Sandwich estimators for logistic and Cox regression were utilized to take into account the hospital-specific clustering of patients.

**TABLE II:** Sex and Age Demographics of Operatively Treated Ankle Fractures in Finland from 1998 to 2020*

	Lateral Malleolar	Medial Malleolar	Bimalleolar or Trimalleolar	Other†
Age *(yr)*	46.4 ± 16.7	42.2 ± 19.8	52.2 ± 17.7	45.4 ± 16.4
Age group				
≤50 yr	17,465 (57.3%)	3,599 (62.7%)	18,901 (43.3%)	2,276 (60.2%)
51-75 yr	11,912 (39.1%)	1,880 (32.8%)	20,813 (47.7%)	1,372 (36.3%)
>75 yr	1,123 (3.7%)	259 (4.5%)	3,936 (9.0%)	130 (3.4%)
Sex				
Men	17,289 (56.7%)	3,410 (59.4%)	17,566 (40.2%)	2,473 (65.5%)
Women	13,211 (43.3%)	2,328 (40.6%)	26,084 (59.8%)	1,305 (34.5%)

* Values are given as the mean ± standard deviation or as the count, with the percentage in parentheses.

† Other includes fibular and/or tibial fractures and unspecified lower-leg fractures (ICD-10 codes S82.4 and S82.9) with a relevant operation code.

## Results

**Fig. 1:**
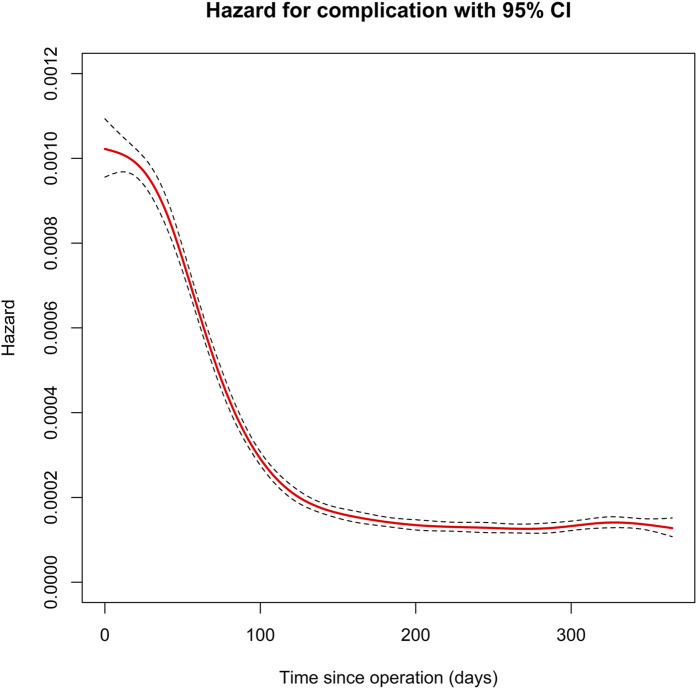
Daily probability of a new complication after ankle fracture surgery, as calculated with use of the estimated hazard function. Solid red line = the probability of a complication, dashed black line = 95% CI.

**Fig. 2:**
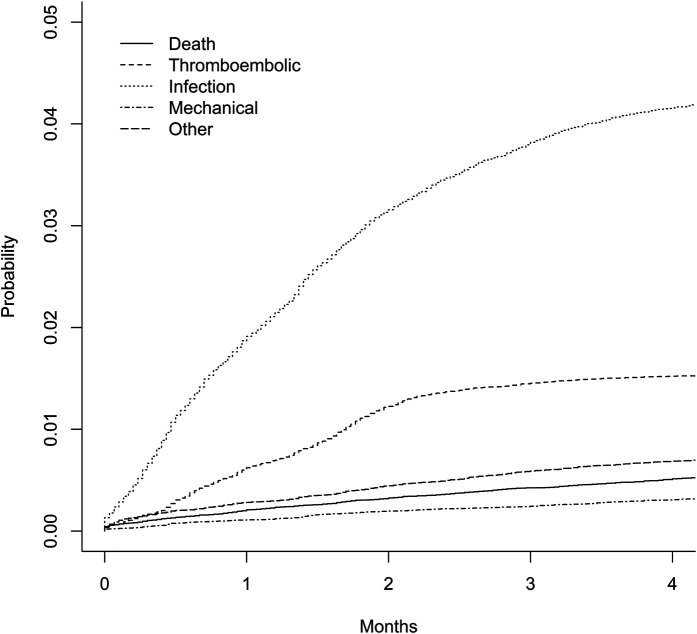
The cumulative probabilities of short-term (≤120 days) complications and death after ankle fracture surgery.

A total of 83,666 ankle fractures were operatively treated between 1998 and 2020. Of these, 36% were lateral malleolar fractures, 7% were medial malleolar fractures, 52% were bimalleolar or trimalleolar fractures, and 5% were other types of fractures. Fifty-one percent of the fractures were in female patients. The mean follow-up time was 10.5 years, and the median follow-up time was 9.9 years. The risk of complications was highest within the first 4 months postoperatively (Fig. 1). The cumulative incidence of short-term complications within the 4-month period was 7.2%. Specifically, infection occurred in 4.4% of cases; thromboembolic complications, 1.6% of cases; mechanical complications, 0.4% of cases; and other complications, 0.9% of cases (Fig. 2). An age of >75 years was associated with a higher rate of short-term complications than an age of 51 to 75 years, with an OR of 1.53 in the multivariable analysis (95% CI, 1.39 to 1.67; p < 0.001). Short-term complications were also more prevalent in patients with diabetes (with or without associated complications); patients with chronic pulmonary, kidney, or liver disease; and patients with peripheral vascular disease (see Appendix Supplementary Table 1). The 4-month mortality rate was 0.6%.

Patients with chronic diseases such as diabetes with complications (OR, 2.26; 95% CI, 1.82 to 2.78; p < 0.001), diabetes without complications (OR, 1.78; 95% CI, 1.51 to 2.10; p < 0.001), chronic liver disease (OR, 1.67; 95% CI, 1.22 to 2.25; p < 0.001), or peripheral vascular disease (OR, 1.67; 95% CI, 1.25 to 2.20; p < 0.001) were more likely to experience an infection than patients without these diseases. Moreover, patients with bimalleolar or trimalleolar fractures were more likely to experience an infection (OR, 1.53; 95% CI, 1.42 to 1.66; p < 0.001) than patients with lateral malleolar fractures, as were patients >75 years old when compared with patients 51 to 75 years old (OR, 1.24; 95% CI, 1.11 to 1.40; p < 0.001).

**Fig. 3:**
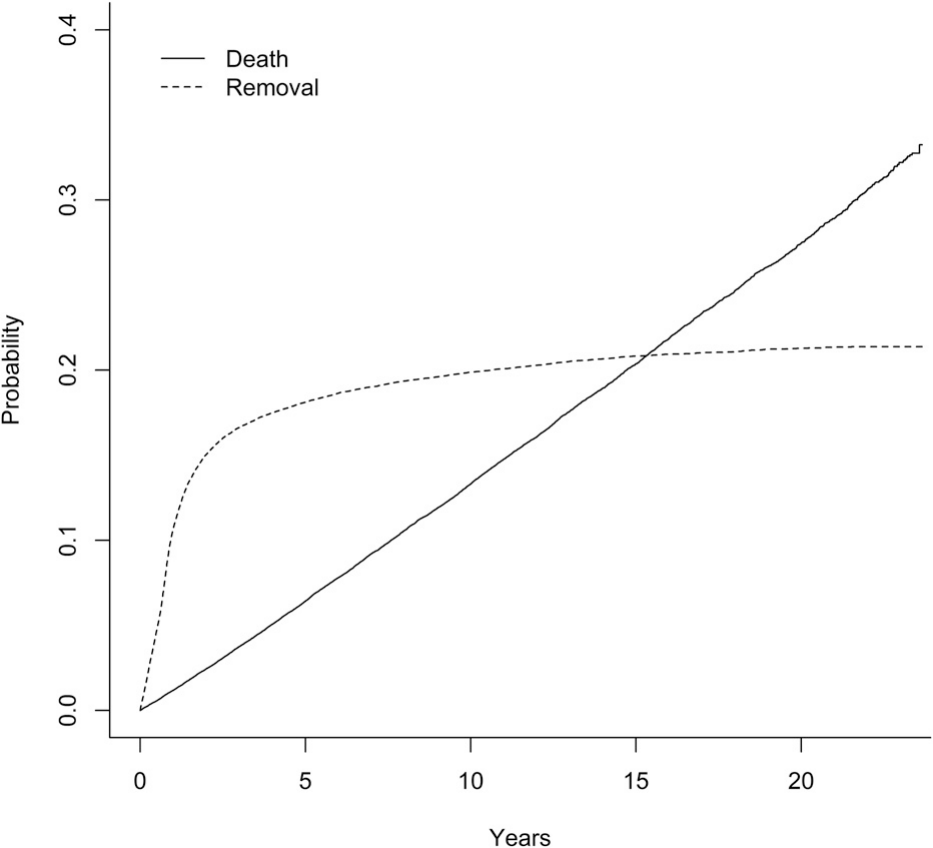
The cumulative probabilities of implant removal and death after ankle fracture surgery.

In the long term, fixation device removal was the most common reason for reoperation. The cumulative incidence of fixation device removal was 11% at 1 year and 17% at 3 years. In the Cox proportional hazards regression models, the risk of fixation device removal increased for patients <50 years old and for patients with bimalleolar or trimalleolar ankle fractures (see Appendix Supplementary Table 2). Mortality was low and increased linearly in the years after the primary fracture surgery (Fig. 3). In the Cox proportional hazards regression models, an age of >75 years, short-term infection, and each of the studied chronic diseases were each associated with an increased risk of death.

**Fig. 4:**
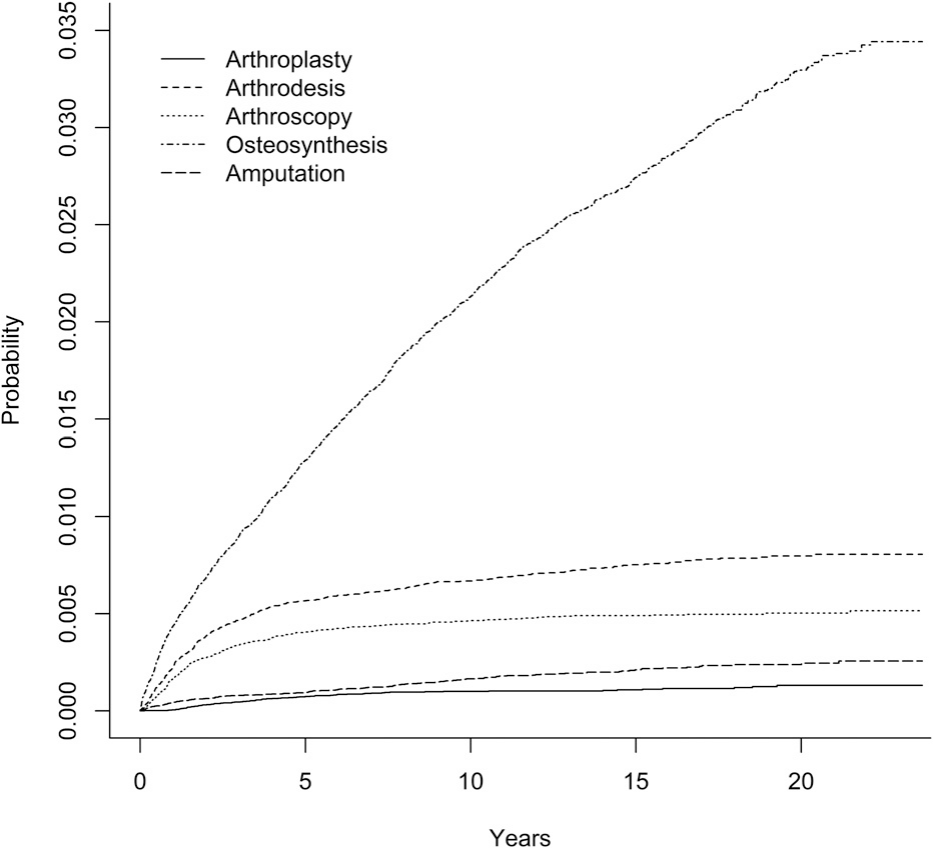
The cumulative probabilities of long-term (>120 days) reoperations after ankle fracture surgery.

The rates of other reoperations and below-the-knee amputations in the long term were low. Specifically, for each procedure type, the rates at 1 year, at 3 years, and at the maximum follow-up (up to 23 years) were as follows: below-the-knee amputation, 0.04%, 0.08%, and 0.3%, respectively; ankle joint arthroplasty, 0.005%, 0.05%, and 0.13%; ankle arthrodesis, 0.2%, 0.5%, and 0.8%; ankle arthroscopy, 0.2%, 0.3%, and 0.5%; and osteosynthesis, 0.4%, 0.9%, and 3.4% (Fig. 4). In the Cox regression models, diabetes with complications, rheumatological disease, short-term infection or a mechanical complication, bimalleolar or trimalleolar fracture, and previous fixation device removal were each associated with a higher probability of reoperation in the long term (see Appendix Supplementary Table 2). These are associations and we could not confirm causation.

## Discussion

Our study, which included all ankle fractures treated operatively in an inpatient or outpatient setting in Finland during a 23-year period, showed that the cumulative incidence of complications needing hospital readmission in the short term (≤120 days) was 7.2%. In the long term (>120 days), the most common reoperation was fixation device removal, with a cumulative incidence of 17% at 3 years after the primary operation.

Ankle fractures are common and often require surgical treatment, and therefore it is essential to be aware of the associated short and longer-term problems. The clinical purpose of our study was to help to identify patients who are prone to complications or reoperations after undergoing operative treatment for ankle fracture. The data were from an unselected, nationwide total population register. The study was comprehensive with a long follow-up. As such, the findings herein can be considered reliable for use in pragmatic clinical decision-making and treatment recommendations. In addition, to our knowledge, our study constitutes the largest cohort of operatively treated ankle fractures published to date and is the first to demonstrate the long-term cumulative incidences of ankle arthrodesis, arthroplasty, and below-the-knee amputation following primary ankle fracture surgery.

Our results align with those of previous studies, supporting and confirming their findings. In a registry study published in 2022, Danilkowicz et al. found that postoperative complications increased linearly with age and that smoking, diabetes, and being overweight increased the risk of complications. They were unable to associate complications with a particular type of fracture. The rate of complications was 5.6%^14^.

Miller et al.^15^ and Ovaska et al.^16^ investigated risk factors for wound complications following ankle fracture surgery. In the study by Miller et al., 1.25% of patients needed revision surgery for wound debridement and 2.9% of patients had a minor complication (i.e., a complication requiring outpatient wound care and/or oral antibiotics in order to achieve complete resolution). The risk factors were diabetes, a known peripheral neuropathy, a history of wound-healing-compromising medications, an open fracture, and postoperative noncompliance^15^. Ovaska et al. reported that the incidence of deep wound infections was 6.8% and that the risk factors for complications were diabetes, alcohol use, fracture dislocation, and associated soft-tissue injuries^16^. In the present study, infectious complications were more common among the elderly, individuals with diabetes, patients with chronic liver conditions, and those with peripheral vascular diseases.

Implant removal can be planned (asymptomatic) or unplanned (symptomatic). The present study could not separate the data by the type of implant removal, but Fenelon et al. reported that 13% of patients who had undergone an operation for ankle fracture had fixation device removal in 10 years. Of these patients, 49% underwent a planned operation (of whom 95% underwent a syndesmotic screw removal), 46% underwent an unplanned removal of symptomatic implants, and 4% underwent an unplanned operation for infection. The median time to surgery was 3 months for planned operations and 13 months for unplanned operations^17^. Because the long-term follow-up period of the present study began 120 days after the primary surgery, we assume that we excluded most of the planned, time-based implant removals, such as those for syndesmotic screws and Kirschner wires. Unplanned implant removal is performed mainly because of fixation-device-related pain, irritation, or infection. We assume that our long-term data primarily represent unplanned implant removals. Additionally, our study showed that, 3 years after ankle fracture surgery, the cumulative incidence of a reoperation for implant removal was 17%. This finding is in line with that of previous studies^18,19^ (Table III).

**TABLE III:** Comparison of Studies of Complications and Reoperations Following Operative Treatment for Ankle Fracture*

First Author	Publication Year	Country	Study Type	Cases	Follow-up Time	Complication and/or Reoperation Rates
Short-term complications						
SooHoo^5^	2009	U.S.	Registry study	57,183	<90 days	Pulmonary embolism, 0.34%; mortality, 1.07%; wound infection, 1.44%; amputation, 0.16%; and revision ORIF, 0.82%
Miller^15^	2012	U.S.	Retrospective cohort study	478	Min. 3 months	Wound complication requiring surgical debridement, 1.25%; wound complication requiring dressing care or a course of oral antibiotics, 2.9%
Ovaska^16^	2013	Finland	Case-control study	1,923	2-52 months	Deep infection, 6.8%
Danilkowicz^14^	2022	U.S.	Registry study	27,633	30 days	Complications, 5.6%, including any readmission, 2.5%, and infection, 1.1%
Happonen	Current study	Finland	Registry study	83,666	120 days	Complications, 7.2%, including infection, 4.4%; thromboembolic complications, 1.6%; mechanical complications, 0.4%; and other, 0.9%
Long-term complications or reoperations						
SooHoo^5^	2009	U.S.	Registry study	57,183	<5 years	Ankle arthrodesis or replacement, 0.96%
Naumann^18^	2016	Norway	Retrospective cohort study	997	Min. 3 years	Implant removal, 17%
Pincus^6^	2017	Canada	Retrospective cohort study	45,444	1 to 2 years	Reoperation, 19.6%. At 1 year: revision ORIF, 1%; reoperation for infection, 0.5%. At 2 years: implant removal, 18.1%
Axelrod^8^	2020	Canada	Retrospective matched cohort study	44,133	Min. 2 years	Ankle arthrodesis or arthroplasty, 0.65%
Fenelon^17^	2019	Ireland	Retrospective study	1,482	2-123 months	Implant removal, 12.5%
Partio^19^	2020	Finland	Registry study	68,865	During a 20-year study period	Implant removal, 27%
Happonen	Current study	Finland	Registry study	83,666	>120 days	Implant removal: 11% at 1 year, 17% at 3 years. Ankle arthrodesis or arthroplasty: 0.23% at 1 year, 0.5% at 3 years, and 0.9% at up to 23 years. Below-the-knee amputation: 0.04% at 1 year, 0.08% at 3 years, and 0.3% at up to 23 years

* ORIF = open reduction and internal fixation.

In 2009, SooHoo et al. reported the findings of their registry study that included patients who had undergone inpatient operations in non-federal hospitals in California between 1995 and 2005^5^. Sixteen percent of fractures were lateral malleolar fractures and 84% were bimalleolar or trimalleolar fractures. The average age was 51 years, and 63% of patients were female. Because SooHoo et al. included only inpatients, their study had a higher mean age and more bimalleolar or trimalleolar fractures than the present study. SooHoo et al. also reported that the rate of intermediate-term complications (<5 years) was low, with ankle arthrodesis or replacement performed in 0.96% of patients. Fracture type was a strong predictor of reoperation for ankle fusion or replacement. In the present study, the 5-year cumulative incidence of ankle arthrodesis and arthroplasty was even lower, at 0.64%.

Axelrod et al. conducted an extensive registry study on the risk of ankle arthrodesis or arthroplasty among patients in Canada who had undergone operative treatment (44,133 patients) or nonoperative treatment (88,266 patients) for ankle fracture^8^. They concluded that, compared with a matched control group, and after adjustment for medical comorbidity, patients with operatively treated ankle fractures had a 3.5-times increased likelihood of undergoing arthroplasty or arthrodesis. In the operative group, 0.65% of patients later underwent arthrodesis or arthroplasty, which occurred at a median of 2.8 and 6.9 years, respectively. Surgical treatment, older age, comorbidity, and post-injury infection significantly increased the risk of later undergoing arthrodesis or arthroplasty. In comparison, in the present study, the 10-year cumulative incidence of ankle arthrodesis and arthroplasty was 0.8%. Furthermore, post-injury infection increased the risk of reoperation (OR, 1.79; 95% CI, 1.57 to 2.04; p < 0.001), but older age decreased the risk of reoperation.

The strengths of our study are the extended follow-up time and the use of data collected from a high-quality, comprehensive national register, which covers the entire patient population of Finland. Furthermore, research has shown that Finnish CRHC data have good coverage and reliability^9,20^. We also took into account the volume of ankle fracture operations in hospitals and controlled for possible secular trends by including the year of the primary operation in the models (see Appendix Supplementary Tables 1 and 2). One limitation associated with the CRHC is the lack of individual clinical data, such as the severity of the injury (other than that indicated by diagnosis and operation codes), the detailed reason for the operation, the identity of the surgeon, and patient-related risk factors, such as body mass index, smoking, or alcohol consumption, that were recorded directly during each discharge. A second weakness is that the Finnish adaptation of the ICD-10 has no dedicated code for bimalleolar and trimalleolar fractures; the codes S82.7 (multiple fractures of the lower leg) and S82.8 (fractures of other parts of the lower leg) are mainly utilized to categorize these types of fractures. Additionally, we included the ICD-10 code S82.9 (fracture of the lower leg, part unspecified) because it was frequently recorded with operation codes that were specific to ankle fracture. Nevertheless, fractures with an S82.9 diagnosis code that lacked a relevant operation code were excluded from our analysis. Another limitation is that complications were identified in the CRHC with use of diagnosis and procedure codes that were consistent with hospital readmissions following the ankle fracture surgery. Although admissions and readmissions are easily linked with use of personal identity codes, there is uncertainty surrounding the identification of complications with relevant diagnoses and procedure codes. In Finland, all major postoperative complications are evaluated and treated in the hospital. However, some minor complications, such as superficial infections or deep venous thrombosis not requiring hospital care, may not have been included in our data if the patient had contacted an outpatient primary care provider instead of the operating hospital and a minor complication was subsequently detected and treated solely in the ambulatory setting.

### Conclusions

Complications after ankle fracture surgery are relatively rare, but their treatment can cause considerable patient morbidity. It is therefore important to identify the patients who are at risk. Our study demonstrated that elderly patients and patients with diabetes, peripheral artery disease, or chronic lung, kidney, or liver disease were prone to short-term complications following ankle fracture surgery. Furthermore, we found that the long-term risk of reoperations due to end-stage ankle osteoarthritis was low.

## Appendix

Supporting material provided by the authors is posted with the online version of this article as a data supplement at jbjs.org (http://links.lww.com/JBJS/H989).
